# The HADES Yield Prediction System – A Case Study on the Turkish Hazelnut Sector

**DOI:** 10.3389/fpls.2021.665471

**Published:** 2021-06-07

**Authors:** Simone Bregaglio, Kim Fischer, Fabrizio Ginaldi, Taynara Valeriano, Laura Giustarini

**Affiliations:** ^1^CREA - Council for Agricultural Research and Economics, Research Centre for Agriculture and Environment, Bologna, Italy; ^2^Ferrero Hazelnut Company, Ferrero Trading Lux, Senningerberg, Luxembourg

**Keywords:** crop simulation model, machine learning, random forest, yield analysis, decision support system

## Abstract

Crop yield forecasting activities are essential to support decision making of farmers, private companies and public entities. While standard systems use georeferenced agro-climatic data as input to process-based simulation models, new trends entail the application of machine learning for yield prediction. In this paper we present HADES (HAzelnut yielD forEcaSt), a hazelnut yield prediction system, in which process-based modeling and machine learning techniques are hybridized and applied in Turkey. Official yields in the top hazelnut producing municipalities in 2004–2019 are used as reference data, whereas ground observations of phenology and weather data represent the main HADES inputs. A statistical analysis allows inferring the occurrence and magnitude of biennial bearing in official yields and is used to aid the calibration of a process-based hazelnut simulation model. Then, a Random Forest algorithm is deployed in regression mode using the outputs of the process-based model as predictors, together with information on hazelnut varieties, the presence of alternate bearing in the yield series, and agro-meteorological indicators. HADES predictive ability in calibration and validation was balanced, with relative root mean square error below 20%, and R^2^ and Nash-Sutcliffe modeling efficiency above 0.7 considering all municipalities together. HADES paves the way for a next-generation yield prediction system, to deliver timely and robust information and enhance the sustainability of the hazelnut sector across the globe.

## Introduction

Turkey is the cradle of hazelnut cultivation and the largest hazelnut producer and exporter in the world (Erdogan, 2018). The history of hazelnut in Turkey originates in the North of Anatolia, along the Black Sea coast, which is a natural habitat of cultivated hazelnuts (*Corylus avellana* L.). About 700,000 ha of land in Turkey are nowadays devoted to hazelnut cultivation (Turkish Statistical Institute, 2020). Due to interannual yield variations, the average annual hazelnut production varies widely (Frary et al., 2019), fluctuating between 400,000 and 800,000 tons (Turkish Statistical Institute, 2020). Nut yield per hectare tends to be lower than in other countries, mainly due to old orchards where agricultural practices are not fully sustainable (Bozoğlu et al., 2019). Nowadays, Turkey supplies more than 65% of the world hazelnut production (Islam, 2018).

Two main distinct production regions are traditionally identified in Turkey, with contrasting characteristics: the Eastern region, which covers the area from the Georgian border to the Central Black Sea coast, including the municipalities of Samsun, Ordu, Giresun, Trabzon, Rize, and Artvin; and the Western region, which comprises the area of Central and Western Black Sea coasts, including the municipalities of Kocaeli, Sakarya, Duzce, Zonguldak, Bartin, Kastamonu, and Sinop. The Eastern region is considered the best suited for hazelnut cultivation for a climatic standpoint, with generally superior quality (Erdogan, 2018). The orchards are usually small (<2 ha), mostly old (>50 years) and agricultural practices generally do not include mechanization. This region is characterized by narrow coastal plains, abruptly rising hills and mountains parallel to the sea, with hazelnut planting extending up to 30 km inland. The flat area is limited, and most orchards are placed on steep hills with shallow soil. In the Western region, the land is relatively flat or with a gentle slope, allowing mechanization. Hazelnut trees are grown in deep and fertile soils and the orchards are well organized, with larger average size and younger trees, leading to a higher average yield than in the East.

According to the Turkish Statistical Institute (TÜİK), the average yield in the Eastern and Western region in 2004–2019 were 0.72 t ha^–1^ and 1.16 t ha^–1^, respectively. Late frosts represent one of the most dangerous abiotic stress factors contributing to yield reduction in Turkey. For example, 2004 and 2014 registered heavy late frost events, which, despite their relatively short duration, severely impacted the national production. Additionally, hazelnut trees are very susceptible to high temperatures with high vapor pressure deficit during ripening causing a reduction in the photosynthetic activity and in turn on hazelnut yield (Girona et al., 1994). A main peculiarity of hazelnut cultivation is biennial bearing. While the mechanism causing a tree to respond with alternate years of relatively high and then low production is still debated, such yearly variation in production challenges the industry in terms of stock capability, logistics and industrial production. Several attempts are currently being tested to dampen this effect and obtain a more constant production (Rossello et al., 2018).

Despite the relevance of this crop for the confectionary industry, no hazelnut yield prediction systems are currently available to meet the different stakeholders’ needs (Kadiyala et al., 2015). These needs range from the identification of appropriate cropping plans and management decisions (Dury et al., 2011), to the evaluation of the cropping systems’ performances across alternative agronomical, socioeconomic and environmental scenarios (Kasampalis et al., 2018). The common background of standard yield prediction systems is the integration of georeferenced informative layers, referred to pedo-climatic conditions and agricultural management practices, into process-based crop simulation models, to simulate the yield variability in a target area at a given spatial resolution (Hartkamp et al., 1999). The flourishing of machine learning techniques in all anthropic activities, including agriculture, is opening new perspectives for crop yield prediction, given the availability of historical datasets to train the models and to validate their performance (van Klompenburg et al., 2020).

Recent years have seen the publication of the first attempts to develop a robust method for hazelnut yield prediction. The HAZEL model presented in Bregaglio et al. (2016) is a process-based yield simulator that reproduces the tree growth and development, simulating the interactions among the main physiological processes and environmental conditions. Building on this paper, Bregaglio et al. (2020) extended the application of HAZEL to four orchards in each of the three countries (Italy, Chile, and Georgia), using three-year experimental datasets. Through statistical techniques, it was proven that model sensitivity slightly varies across environments, without changes to the ranking of the model parameters. This suggested a good reliability of the obtained results for further applications.

This paper presents an operational prototype of a hybrid model for hazelnut yield prediction, named HADES (HAzelnut yielD forEcaSt), in which process-based modeling and machine learning techniques are integrated. In Materials and Methods, we introduce the datasets required as input, with a statistical analysis of hazelnut yields, and then describe the different HADES modules and the machine learning layer. The Results section reports a quantitative assessment of HADES performance in calibration and validation. In Discussion we examine the advantages and drawbacks of the proposed hybrid system, with an outlook to further research that needs to be conducted, while the Conclusions section deals with the transferability of HADES to an operational setting.

## Materials and Methods

### HADES Workflow

**FIGURE 1:**
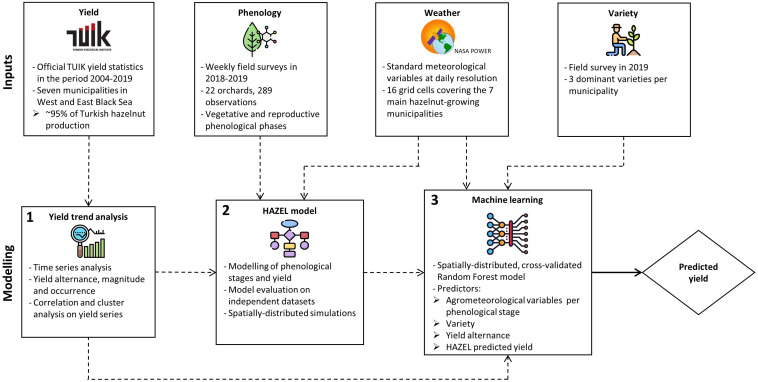
The HADES yield prediction system. Input data sources (official yields, phenological observations, weather data, and main varieties per municipality) are used in a three-step workflow: a statistical analysis of the yield series, the application of the process-based HAZEL model, and the use of a machine learning layer to optimize the system accuracy.

The workflow of HADES is shown in Figure 1. Four sources of input data were used: (i) official statistical hazelnut yield data in the period 2004–2019, as provided by the Turkish Statistical Institute (TÜİK) (Turkish Statistical Institute, 2020), (ii) phenological observations collected in field surveys in 2018--2019 in the main Turkish hazelnut growing municipalities (Sakarya, Duzce, and Zonguldak in the Western Black Sea region; Samsun, Giresun, Ordu, and Trabzon in the Eastern Black Sea region), (iii) information on the three main hazelnut varieties in each municipality collected in a field survey carried out in 2019, and (iv) daily weather data derived from the NASA POWER database^1^. These inputs were used to perform a three-step analysis. In the first step, the statistical analysis of official yields allowed identifying time trends and evaluating the presence and strength of the alternate bearing. This analysis was meant to characterize specific properties of the yield series, which were then used as input to the second step, i.e., the calibration and evaluation of the HAZEL simulation model (Bregaglio et al., 2016). Here, the NASA POWER weather data were used as inputs, while the phenological observations were employed as reference data to derive a single parameter set for all the Turkish hazelnut growing regions. This was needed to calibrate HAZEL, aiming at maximizing its accuracy in predicting TÜİK official yields. The third and last step of the HADES workflow is a Random Forest (RF, Breiman, 2001) regression-based machine learning layer, which uses as input the three main hazelnut varieties grown in each municipality, the yields predicted by HAZEL (step 2), several agro-meteorological indicators, and the outputs of the yield analysis (step 1). The final accuracy of HADES in predicting official yield was assessed as the median performance in model validation, over all 100 bootstrap samples drawn with replacement from the original input datasets.

### Statistical Analysis of Official Yield Data

Data on hazelnut production (t) and cultivated area (dekare) in the period 2004–2019 were downloaded from the data portal of the Central Dissemination System of TÜİK at NUTS3 (municipality) level. Yield, expressed in t ha^–1^, was derived as the ratio between production and cultivated area for the seven municipalities of Duzce, Sakarya and Zonguldak in the Western part of Turkey, and Giresun, Ordu, Samsun and Trabzon in the Eastern part of the country. The choice of the municipalities was driven by their relevance in term of cultivated area, given that they account for 95% of the total average hazelnut area in the considered period.

Basic statistics (mean and standard deviation) were calculated at the municipality level, and a trend analysis (Siegel, 1982) followed by a Mann-Kendall test (Mann, 1945; Kendall, 1975) were performed to identify any linear trends in the series and to assess the significance of the resulting slopes. Similarities in yield trends between municipalities were evaluated using Pearson’s correlation and allowed to cluster municipalities, using complete linkage as clustering criterion and Euclidean distance as the distance metric.

Two indices were used to characterize the occurrence and magnitude of alternate bearing in the yield time series, as proposed by Hoblyn et al. (1937). Occurrence was evaluated using the bienniality index (B), which firstly identifies the sign of variation in annual yields, and then quantifies the percentage of occurrence of the typical on-off pattern (min: 0%—no biennial bearing, max: 100%—perfect alternance). The intensity of fluctuation was computed as:

(1) $$I=\sum_{i=2}^{n}\frac{\left|y_{i}-y_{i-1}\right|}{y_{i}+y_{i-1}}/\left(n-1\right)$$

where I is the sum of the absolute difference between two consecutive yields (y_*i*__–__1_ and y_*i*_) divided by their sum and averaged by the number of data points (n). I = 1 corresponds to maximum alternate bearing with no hazelnut yield in the off year, whereas I = 0 indicates constant yield (no alternate bearing) in the series. The significance of the I value was tested after recalculating I on synthetic yield series, derived by bootstrap resampling with replacement (5,000 samples) of the original data series, and considering the frequency of exceedance of the original I value (Huff, 2001). The higher the frequency, the lower the likelihood of alternate bearing.

A new method has been developed to label annual yields as on/off years. The procedure is based on the computation of a yearly index I^∗^, derived from I, which considers both the sign and the intensity of the variation in annual yield, and is used to classify each year with respect to the previous one.

(2) I=*yi-yi-1yi+yi-1

A positive value of I^∗^ indicates an on year and a negative value indicates an off year, provided that the intensity of variation is significant (Eq. 3). Yield variation was considered negligible when its absolute value (| I^∗^|) was lower than 99.9% of the I values obtained from the permutations of the whole series; under this threshold, indicated as I_0.001_, biennial bearing does not occur in yield data series, which can in turn be considered flat and constant. Yearly yield data were then labeled as unexpected when they have the same label as the previous year.

(3) y⁢e⁢a⁢r⁢l⁢a⁢b⁢e⁢l={o⁢nif(|I|*>I0.001)∧(I>*0) o⁢f⁢fif(|I|*>I0.001)∧(I<*0) p⁢r⁢e⁢v.y⁢e⁢a⁢r⁢l⁢a⁢b⁢e⁢lo⁢t⁢h⁢e⁢r⁢w⁢i⁢s⁢e 

### HAZEL Model Calibration and Evaluation

HAzelnut yielD forEcaSt is a process-based simulation model that reproduces the effect of seasonal environmental conditions on the phenological development and growth dynamics of a hazelnut tree (Bregaglio et al., 2016). The simulation run starts at the end of the previous cropping season, set to September 1 in Turkey, when a sequential phenological model starts to be executed with separate chilling (Črepinšek et al., 2012) and degree day accumulation. Chilling hours accumulate when air temperatures are between 0 and 7°C. Female flowers are receptive to pollen in mid-winter and leaf budbreak occurs in the spring. The phenological model of hazelnut vegetative and reproductive phases presented in Bregaglio et al. (2016, 2020) was updated according to the new assessment scale in use by the agronomists, who performed the field observations (Supplementary Table 1.1). Ten phenological phases are modeled for vegetative development, from dormant buds to leaves dropping, whereas reproductive stages are separated for catkins (male flowers) and female inflorescences, from flowering to nuts dropping. Initial leaf area index (LAI) is then assigned according to plant dimensions: plant height and crown size. The light interception model considers the inter- and intra-row distance (Pronk et al., 2003) and uses as input the direct and diffuse light components, which are derived from global solar radiation (Spitters et al., 1986). Plant gross photosynthesis is simulated with a decoupled stomatal conductance (Jarvis, 1976) and carbon assimilation model (Chen et al., 1999). Net assimilation is derived considering the losses due to maintenance and growth respiration of leaves, fruits, stem, branches and fruits (de Vries et al., 1989). The partitioning to tree organs is simulated as dependent on phenological phases, with hazelnut growth starting from the development of the ovary (R10 in Supplementary Table 1.1). At the end of each day, the growth rate of green LAI is computed from specific leaf area and the rate of biomass partitioned to the leaves. Full algorithmic description is provided in Bregaglio et al. (2016). The impact of late frost was simulated according to the algorithm presented in Supplementary Material 2, considering the increasing susceptibility of hazelnut trees from female flowering to ovary enlarging, based on experimental work from Chozinski (1995). The alternate bearing pattern was reproduced by reducing the maximum daily portion of assimilates partitioned to fruits in off years according to a municipality-specific coefficient, corresponding to the average percentage reduction between on and off years, derived from the statistical analysis of official yields (section 2.2).

Model calibration focused first on phenology, using field observations collected in 2018 and 2019 on 22 sites located in the main hazelnut producing municipalities, as reference data (total 289 observations). The thermal thresholds to reach the hazelnut vegetative and reproductive phases were adjusted using a multi-start simplex automatic optimization algorithm, according to Bregaglio et al. (2020), to give a single parameter set representative for the entire Turkish hazelnut region. Then, the most relevant parameters, selected according to the sensitivity analysis of Bregaglio et al. (2020), were calibrated using the same optimization algorithm, separately for Western and Eastern municipalities. Model evaluation was performed using the hold-out method stratified by municipality (7 classes) and alternate bearing (on-off, 2 classes). The original yield dataset was split in two equal parts, one for calibration activities and the other for evaluation of model performance on independent data (Klemes, 1986), making sure that each subset maintains the same proportions of classes as the total dataset (Kohavi, 1995). Parameter values after calibration are presented in Supplementary Material 1.

The daily minimum and maximum air temperature (°C) used as input for the simulations was downloaded from the NASA POWER database, which provides gridded data at 0.5° resolution for the entire globe. We selected 17 grid cells covering the hazelnut growing regions in the seven selected municipalities. Daily global solar radiation (MJ m^–2^ d^–1^) as input to HAZEL was estimated from air temperature according to Hargreaves and Samani (1982).

### Machine Learning Layer

Besides process-based simulation models, machine learning techniques have opened new perspectives in crop yield prediction. The machine learning layer in HADES relies on RF regression, where many regression trees are grown without pruning, using random bootstrap samples of the input data, and all trees are averaged to come up with the conclusive response. The ranking of the importance of individual predictor variables is computed as the increase of mean squared error (MSE) in the prediction of internally held-out samples, resulting from the permutation of the respective predictor in the model (Breiman, 2001; Liaw and Wiener, 2002). The higher this increase in MSE after permuting the predictor, i.e., after breaking its original relationship with the response variable, the greater its importance. To create the response variable for RF regression, the original TÜİK yield time series data were split into a calibration (75% of the overall data; *n* = 84) and a validation dataset (25% of the overall data; *n* = 28) stratified by municipality, so that each municipality appeared exactly four times in the validation set, regardless of the growing season. Each split represents one bootstrap sample. In order to come up with robust metrics of HADES accuracy in predicting official yield, 100 bootstrap samples with replacement were created from the original yield series. Furthermore, to avoid model overfitting and to retrieve more reliable accuracy metrics, leave-one-out cross validation was performed during model training. The final model accuracy, evaluated in terms of relative root-mean-square error (RRMSE), mean absolute error (MAE), coefficient of determination (R^2^), Nash–Sutcliffe model efficiency (EF) and coefficient of residual mass (CRM), was determined as the median values of the respective metric in model validation over all 100 bootstrap samples. Following the same logic, the final variable importance ranking was assessed as the median value of percentage increase in MSE over all bootstrap samples. In order to evaluate spatial patterns in yield prediction, maps for each growing season showing the percentage error in prediction per municipality were created.

Four different sources of input variables were used as predictors: (i) the three main hazelnut varieties grown in each of the major hazelnut growing municipalities, (ii) the alternate bearing pattern in terms of labels (on/off) for each growing season as derived from the statistical analysis of official yield series (section 2.2), (iii) the yields per municipality in each growing season as predicted by HAZEL (section 2.3) and (iv) agro-meteorological indicators computed according to the phenological development in each municipality and growing season. In order to compute the agro-meteorological indicators at the municipality level, daily weather data from the same 17 NASA POWER grid cells used as input to HAZEL were aggregated. For a subset of 14 phenological phases (R7—R13; V1—V7, Supplementary Material 1) per municipality and growing season, the following predictors were calculated: minimum, average and maximum temperature (°C), total precipitation (mm), relative humidity (%) and maximum wind speed (km h^–1^). This sums up to a total of 89 predictors (3 main varieties, on/off label, yield predicted by HAZEL, 14 phenological phases × 6 indicators). The yield predictions were simulated at the end of each growing season (August 31), and only weather data before this date were incorporated into the RF model, i.e., no weather forecast was used in the set of predictors. The RF machine learning layer of HADES was developed in the R free statistical software (R Core Team, 2020) using the “caret” package (Kuhn, 2008), which internally relies on the widely used “randomForest” package (Liaw and Wiener, 2002).

## Results

### Analysis of Official Yields in Turkey by Region and Municipality

**FIGURE 2:**
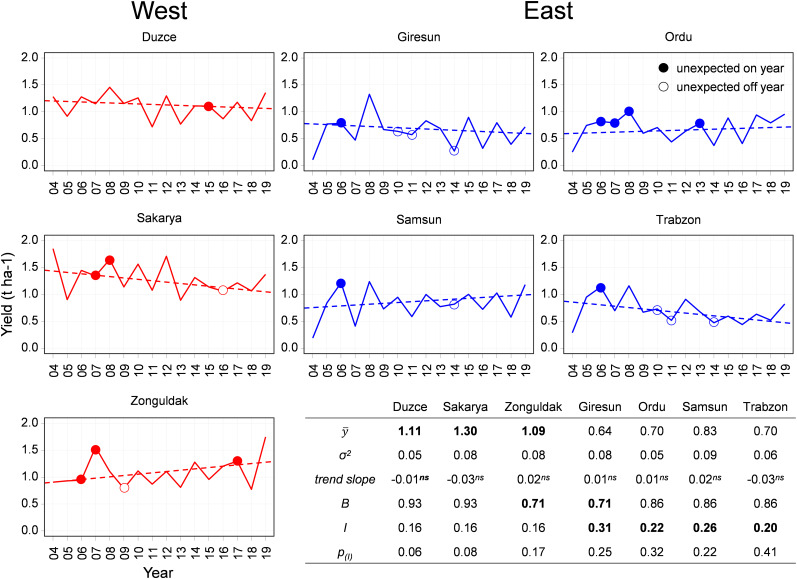
The plots show yield data in seven Turkish municipalities (red continuous line for Eastern municipalities, blue for Western municipalities) in the period 2004–2019. Unexpected on and off years are marked with a circle. The yield trend is reported as a dashed line. The table shows the statistics of the series: mean yield ($\bar{y}$) and variance (σ^2^), trend slope, bienniality index (B), alternate bearing intensity (I) and significance.

The statistical yield analysis performed in the seven main hazelnut growing municipalities is presented in Figure 2.

No significant time trend (*p* = 0.05) was detected in the official yield time series. The average yield in the period 2004–2019 in the Western municipalities was higher than in the Eastern region, with Sakarya ($\bar{y}$ = 1.3 t ha^–1^) ranked as the municipality with the highest yield, followed by similar values in Duzce ($\bar{y}$ = 1.11 t ha^–1^) and Zonguldak ($\bar{y}$ = 1.09 t ha^–1^). In the Eastern region, Giresun was the municipality with the lowest yield ($\bar{y}$ = 0.64 t ha^–1^), whereas Ordu and Trabzon were characterized by the same average yield ($\bar{y}$ = 0.7 t ha^–1^), and eventually Samsun emerged as the highest yielding municipality ($\bar{y}$ = 0.83 t ha^–1^). Standard deviations were very similar across municipalities, with values ranging from 0.05 t ha^–1^ in Duzce and Ordu to 0.09 t ha^–1^ in Samsun.

The characterization of the occurrence of alternate bearing in the series, quantified *via* the bienniality index (B), highlighted that the majority of annual yield data in all municipalities followed the expected on-off pattern. The percentage of years in which alternate bearing was detected ranged between 71% in Zonguldak and Giresun, to 93% in Duzce and Sakarya. After bootstrap resampling with replacement to quantify the intensity of alternate bearing, the calculation of the I index led to higher values in the Eastern region, ranging from 0.20 in Trabzon to 0.31 in Giresun, where yield variations were the highest. A slightly lower value (0.16), but with higher significance was computed in all the Western municipalities (Supplementary Material 3). According to the procedure developed to highlight anomalies in the yield series (section 2.2), the following years were labeled as unexpected on year: 2006 in Zonguldak in all Eastern municipalities, 2007 in Zonguldak, Sakarya and Ordu, 2008 in Sakarya and Ordu, 2013 in Ordu and 2015 in Duzce. Few cases of unexpected off years were detected in the Western region (2009 in Zonguldak and 2016 in Sakarya), whereas in the Eastern region the years 2010 and 2011 in Giresun and Trabzon, and the year 2014 in Giresun, Trabzon and Samsun were labeled as anomalies.

**FIGURE 3:**
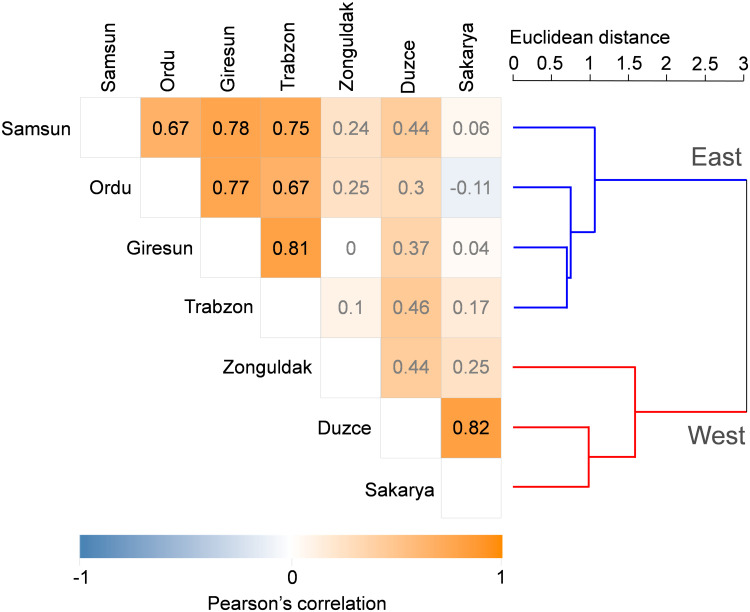
Pearson correlation matrix (on the **left**) and cluster analysis (on the **right**) on the yield data in the seven main hazelnut growing municipalities. Non-significant correlation values are reported in gray.

The correlation and the cluster analyses performed on the official yield series in the seven main hazelnut producing municipalities are graphically presented in Figure 3. The highest positive correlation between yield data series was found between Sakarya and Duzce (Pearson *r* = 0.82, *p* < 0.05), followed by Giresun and Trabzon (*r* = 0.81, *p* < 0.05). The correlations between Zonguldak yield data and the other municipalities were positive but not significant. High positive correlations (*p* < 0.05) were also found between the yield data corresponding to the Eastern municipalities, with a decreasing strength from the pair Samsun-Giresun (*r* = 0.78) to the following ones: Giresun-Ordu (*r* = 0.77), Samsun-Trabzon (*r* = 0.75), Samsun-Ordu and Ordu-Trabzon (*r* = 0.67). The application of the clustering algorithm led to a clear distinction between the Eastern and Western region and was used as the criterion to develop two distinct parameter sets of the HAZEL model. The Euclidean distances computed between the yield data series were 1.06 and 1.59 for the Eastern and Western municipalities, respectively. The Euclidean distance between the entire yield data series was 3.04.

### Process-Based Model Calibration and Evaluation

A synthetic overview of HAZEL performance in reproducing phenological development and yield in calibration and evaluation datasets is provided in Table 1.

**TABLE 1:** HAZEL model performance in reproducing hazelnut phenology, considering catkin development, female flowers/fruits ripening and vegetative phase and yield.

**Region**	**Variable**	**Unit**	**Period**	**Use**	**Data**	**MAE**	**RRMSE**	**EF**	**CRM**	**R^2^**
All municipalities	Catkin development	days	2018–2019	C	70	15.5	–	0.98	–0.01	0.98
	Female flowers/fruits	days	2018–2019	C	112	11.1	–	0.96	0.02	0.96
	Vegetative phases	days	2018–2019	C	87	15.6	–	0.86	–0.09	0.91
	Yield	t ha^–1^	2004–2019	C	88	0.19	28.6%	0.55	–0.11	0.65
				E	88	0.16	27.2%	0.56	–0.11	0.68
East	Catkin development	days	2018–2019	C	45	18.4	–	0.98	–0.01	0.98
	Female flowers/fruits	days	2018–2019	C	61	10.5	–	0.96	–0.04	0.96
	Vegetative phases	days	2018–2019	C	47	21.5	–	0.76	–0.14	0.85
	Yield	t ha^–1^	2004–2019	C	47	0.19	34.5%	0.41	–0.09	0.48
				E	49	0.13	26.4%	0.59	–0.04	0.62
West	Catkin development	days	2018–2019	C	25	10.1	–	0.99	–0.02	0.99
	Female flowers/fruits	days	2018–2019	C	51	11.9	–	0.97	0.09	0.98
	Vegetative phases	days	2018–2019	C	40	8.6	–	0.96	–0.01	0.98
	Yield	t ha^–1^	2004–2019	C	41	0.19	22.6%	0.49	–0.11	0.61
				E	39	0.19	23.8%	0.25	–0.13	0.51

*Statistical metrics quantifying model accuracy are synthesized for all main hazelnut growing municipalities and separately for Eastern and Western region. C, calibration dataset; E, evaluation dataset; MAE, mean absolute error; RRMSE, relative root mean square error; CRM, coefficient of residual mass.*

The phenological observations collected in 2018 and 2019 growing seasons were reproduced by HAZEL with variable accuracy. The reproductive phases related to female flowers and fruits (112 observations) were simulated with a MAE of 11.1 days (11.9 days in Western and 10.5 days in Eastern regions), with a slight overestimation in the East (CRM = −0.04) and the opposite in the Western region (CRM = 0.09) (Table 1). The MAE committed by the model in simulating catkin development (70 observations) and vegetative phase (87 observations) was larger (15.5 and 15.6 days, respectively), the latter being better reproduced in the Western (8.6 days) than in the Eastern region (21.5 days). Regarding yield simulations, overall model performances in calibration and evaluation were very similar (Table 1), with less than 0.2 t ha^–1^ of MAE, with overestimation (CRM = −0.11). On average, model accuracy according to the RRMSE was higher in the Western region (less than 24.0% RRMSE in calibration and evaluation) than in the East, where RRMSE in calibration reached 34.5%. EF values ranged between 0.49 and 0.59 in Eastern and Western region, respectively. The correlation between simulated and official yields was always statistically significant at *p* = 0.01, with overall R^2^ value higher than 0.65, and ranging between 0.48 and 0.62 in calibration and evaluation when considering the two regions (Table 1).

**FIGURE 4:**
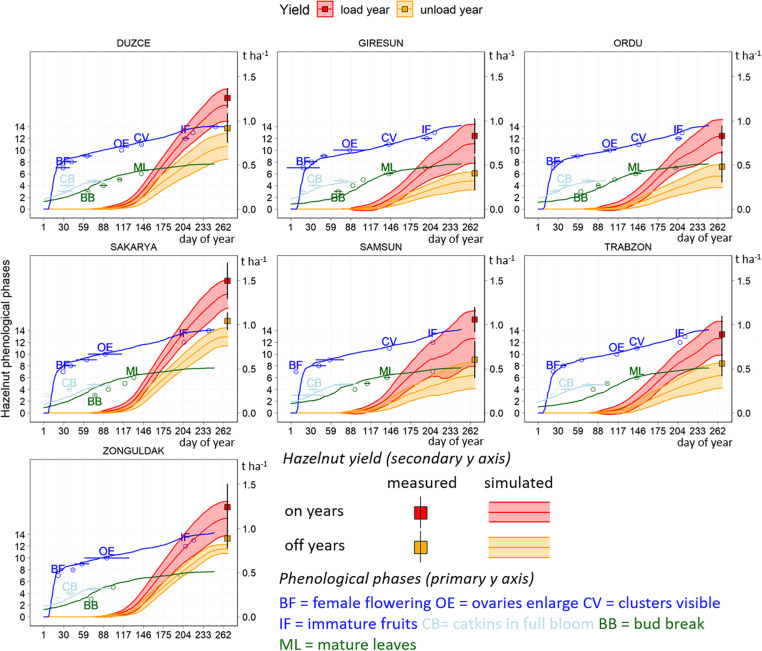
Simulated (lines) and observed (points) dynamics of hazelnut phenology, distinctly presented for catkins (cyan), female (blue) and vegetative (dark green) phases. Simulated data refers to the average model outputs in 2004–2019, observed data to the field samplings collected in 2018 and 2019. Simulated dynamics of hazelnut yield are reported for on (red) and off (orange) years, along with official yield data (squares). Shades and horizontal and vertical error bars correspond to ± one standard deviation.

The simulated dynamics of hazelnut development and yield in the calibration and evaluation datasets are presented in Figure 4, where key phenological phases are reported as compared with field observations in 2018–2019. Simulated and official yield data are presented divided into on and off years, and reporting the associated variability as ± one standard deviation.

Simulations of key hazelnut phenological phases in 2004–2019 were in line with observations. Average dates of beginning of female flowering in field observations spanned between day of year (DOY) 17 (January 17, East) and 28 (January 28, West), with maximum anticipation of 15 days in the West (Figure 4). The start of the ovary development occurred on average on DOY 96 (April 6) in all municipalities in 2018 and 2019, and model results were close to this date in the West, whereas in the East this phase was simulated on DOY 119 (April 29). Nuts clusters were visible around DOY 144 (May 24) in field samplings and were simulated on average on DOY 137 (May 17). The dates when immature fruits were observed and simulated ranged between DOY 198 and 203 (July 17–22), respectively (Figure 4).

Hazelnut yield dynamics started from ovary enlarging and followed a logistic shape, with inflection point close to immature fruits stage, and then with a smoothed increase until harvest. Average official yields in on years ranged between 0.83 t ha^–1^ in Giresun and Ordu to 1.06 t ha^–1^ in Samsun in the Eastern regions, with larger variability in Giresun and Trabzon (sd 0.20 t ha^–1^). Corresponding average simulated yields were slightly lower than the official ones and ranged between 0.74 t ha^–1^ in Giresun and 0.85 t ha^–1^ in Samsun (Figure 4). The associated variability was comparable to official data and followed an increasing gradient from Ordu (0.19 t ha^–1^) to Giresun (0.22 t ha^–1^) and Trabzon (0.31 t ha^–1^). In the Western municipalities, average official yields in on years were higher than in the East and ranged between 1.24 t ha^–1^ in Zonguldak (sd 0.26 t ha^–1^) and 1.49 t ha^–1^ in Sakarya (sd 0.20 t ha^–1^). The model reproduced the high yield in on years in Sakarya, although with a slight underestimation (1.36 t ha^–1^, sd 0.16 t ha^–1^). It also replicated the lowest productivity of Zonguldak (1.12 t ha^–1^, sd 0.19 t ha^–1^). Official yields in off years were lower in the Eastern municipalities, comprised between 0.40 t ha^–1^ in Giresun (sd 0.19 t ha^–1^), 0.48 t ha^–1^ in Ordu (sd = 0.13 t ha^–1^), 0.55 t ha^–1^ in Trabzon (0.14 t ha^–1^) and 0.60 t ha^–1^ Samsun (sd 0.21 t ha^–1^). Simulations reproduced the same trend, once more with underestimation ranging from the lowest value in Giresun (0.32 t ha^–1^, sd 0.10 t ha^–1^) to the highest in Samsun (0.43 t ha^–1^, sd 0.15 t ha^–1^). The official and simulated yields were higher in the West, in off years, with Sakarya as the municipality with the highest yield both in the official (average 1.05 t ha^–1^, sd 0.10 t ha^–1^) and simulated data (average 0.86 t ha^–1^, sd = 0.10 t ha^–1^).

### Integration of the Machine Learning Layer

HAzelnut yielD forEcaSt model performance is reported in Table 2, both at the level of the seven main hazelnut growing municipalities and separately for the Western and Eastern regions.

**TABLE 2:** HADES performance in predicting official yields.

**Region**	**Use**	**MAE (t ha^–1^)**	**RRMSE (%)**	**EF (−)**	**CRM (−)**	**R^2^ (−)**
All municipalities	C	0.15	19.78	0.73	0.00	0.72
	V	0.15	19.84	0.73	0.00	0.75
East	C	0.14	23.39	0.60	–0.05	0.62
	V	0.14	23.40	0.60	–0.04	0.62
West	C	0.15	16.72	0.49	0.04	0.51
	V	0.15	16.80	0.48	0.03	0.50

*Statistical metrics quantifying model accuracy are synthesized for all main hazelnut growing municipalities and separately for East and West. C, Cross-validated performance in calibration; V, Median model performance in validation; MAE, mean absolute error; EF, Nash-Sutcliffe modeling efficiency; RRMSE, relative root mean square error; CRM, coefficient of residual mass.*

While the official TÜİK yields were predicted with a MAE of 0.15 t ha^–1^ on the level of the main hazelnut growing municipalities, as well as separately for the Western region, the MAE in the Eastern region was slightly lower (0.14 t ha^–1^). Model performance has proven to be robust with similar MAE values both in leave-one-out cross-validated calibration and in validation. In terms of this metric, model performance improved from HAZEL (Table 1) to HADES (Table 2) for all spatial aggregation levels, both in calibration and validation, with the only exception of the Eastern region in validation, where the MAE value slightly increased (from 0.13 t ha^–1^ to 0.14 t ha^–1^). Model accuracy according to the RRMSE was better in the West (16.76%) than in the East (23.4%). When comparing the RRMSE values before (Table 1) and after (Table 2) the incorporation of the machine learning layer, a significant improvement can be observed at all spatial levels and both in calibration and validation. EF was always positive, with the best result on the level of all municipalities (0.73), followed by the Eastern (0.60) and the Western region (0.49). In terms of CRM, HADES was slightly biased toward overestimating official yields in the East (CRM = −0.05 in calibration and −0.04 in validation), whereas the opposite held true in the West (CRM = 0.04 and 0.03, respectively). Consequently, on the level of all municipalities, the HADES yield estimate was unbiased (CRM = 0.00). R^2^ values for all main hazelnut growing municipalities were 0.72 in calibration and 0.75 in validation, respectively. Considering the two regions separately, values ranged between 0.50 (validation West) and 0.62 (calibration and validation East). Just as EF and CRM, also the R^2^ values improved from HAZEL to HADES, with the only exception of the Eastern region in validation, where R^2^ slightly decreased. A detailed graphical comparison of the model performance on the level of the main hazelnut growing municipalities by means of R^2^ and MAE sampling distributions that resulted from predicting official yields over all 100 bootstrap samples is presented in Supplementary Material 4.

**FIGURE 5:**
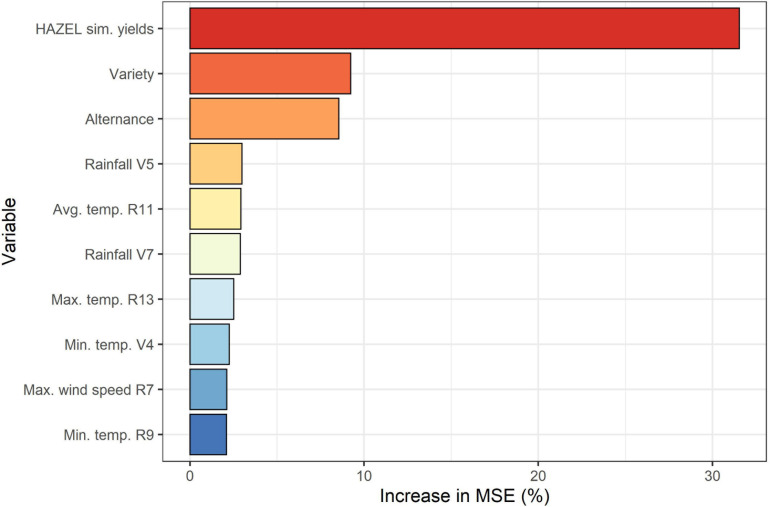
Median variable importance calculated over all 100 model iterations in terms of mean percentage increase in Mean Square Error (MSE, %).

Figure 5 presents the importance of the predictors used in RF regression, determined using the percentage increase of MSE when a respective predictor was randomly permuted.

The yields simulated by HAZEL (section 3.2) emerged as the most important feature, which confirms both that HAZEL’s accuracy in predicting yield is sufficiently high and also that the coupling of a process-based model with a machine learning layer is beneficial to predict official yields. Information obtained in the field, i.e., the three most important hazelnut varieties per municipality, ranked second, underlining the value of ground-based observations in a machine learning-based yield prediction framework. Alternance, which constitutes the outcome of step 1 followed on rank three. Several agro-meteorological variables computed according to the phenological development in each municipality were then ranked from position 4 downward. These remaining features all presented very similar increases in MSE.

**FIGURE 6:**
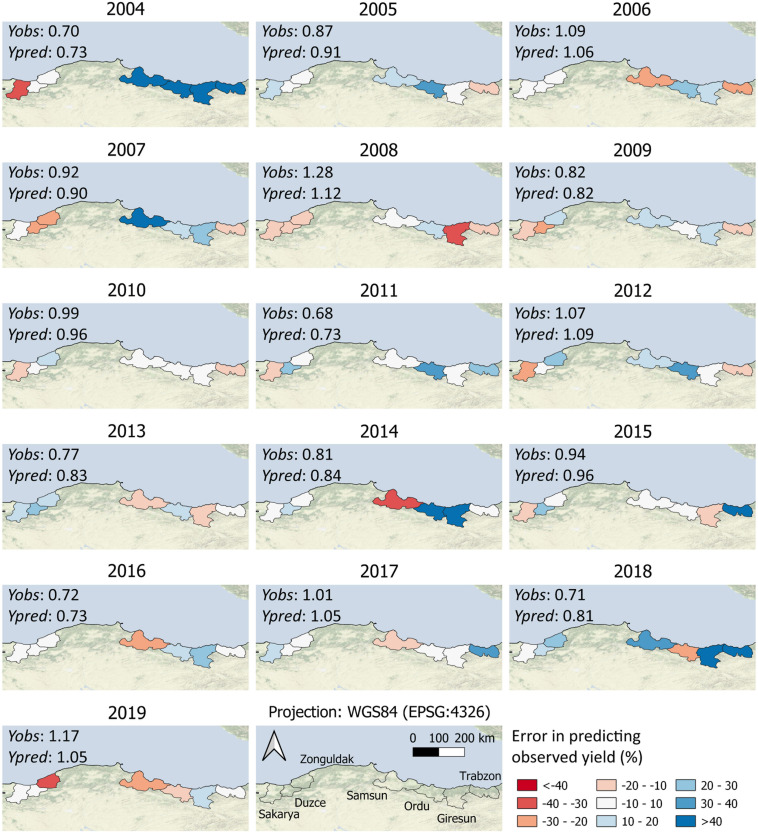
Median model results in terms of percentage error in predicting official yield per municipality and year. The maps report the comparison between absolute values of official (*Yobs*) and predicted yield (*Ypred*).

Figure 6 depicts a detailed spatial overview of the performance of HADES in predicting official yield in the main hazelnut growing municipalities, considering all growing seasons from 2004–2019. Considering all municipality × growing season combinations, HADES obtained absolute percentage errors in the range of ± 10% in 33% of the cases and in the range of ± 20% in 65% of cases. In the Western region, there were only two cases with prediction errors larger than 40%: in Sakarya in 2004 (Yobs = 1.85 t ha^–1^, Ypred = 1.27 t ha^–1^) and in Zonguldak in 2019 (Yobs = 1.75 t ha^–1^, Ypred = 1.21 t ha^–1^). Overall, the percentage errors were higher in the Eastern region, especially in seasons with low yields (e.g., 2004, 2014). In 2004, HADES largely overestimated yields in all Eastern municipalities, even though predicted yield was the lowest of all growing seasons (mean Yobs in the East = 0.21 t ha^–1^, mean Ypred = 0.45 t ha^–1^). The same holds partially true for 2014, where HADES overestimated yields in Ordu and Giresun, while it underestimated it in Samsun (mean Yobs in the East = 0.48 t ha^–1^, mean Ypred = 0.52 t ha^–1^).

## Discussion

### Alternate Bearing Emerged in Hazelnut Yield Series

The subdivision of the Turkish Black Sea coast into an old and a new hazelnut production region is well established in the literature (Islam, 2018). The former covers about 70% of the total hazelnut area and is located in the Eastern Black Sea area, while the latter extends in the Western Black Sea area, comprising the municipalities of Duzce, Sakarya and Zonguldak. However, less information is available on the inter-annual variability of Turkish hazelnut yields, even though it is well known that this tree crop has a marked alternate bearing in most growing environments (e.g., Italy, Roversi and Ughini, 2006; Chile, Ascari et al., 2020; United States, Mehlenbacher et al., 2011). This phenomenon has multiple causes, encompassing biochemical, physiological, genetic and environmental factors (Sharma et al., 2019), and consists of two major multiannual reproductive strategies, leading to heavy fruit load in 1 year, and low fruit load in the following season (Goldschmidt, 2013). Other than providing a quantitative assessment of the yield trends in the main hazelnut producing municipalities through correlation and cluster analyses on official yield data, our study provides the first statistically-based assessment of the frequency and strength of the alternate bearing in Turkish hazelnut yields. This study confirmed that the year-to-year yield fluctuations can be mostly explained by a biennial pattern in the examined period (2004–2019), and that their magnitude strongly differs among hazelnut growing municipalities. The elaboration of a new index to understand the outliers of the expected on-off sequence allowed identifying anomalies in the yield data series: the cause of such outliers requires a dedicated investigation in the future. As a preliminary explanation, it needs to be highlighted that the exceptionally low yield in 2004 and the occurrence of the unexpected off year in 2014 in the Eastern municipalities were probably associated with late frost events which caused serious damage to the yield (Erdogan and Aygun, 2017; An et al., 2020). The cold stress function implemented in the HAZEL model (Supplementary Material 2) allowed capturing these events, as well as the occurrence of cold stress in 2012, which lays the basis for an extended evaluation of the sensitivity of hazelnut trees to cold stresses, based on site-specific weather, phenological and yield data. This would potentially allow the definition of a methodology to be used in operational forecasting activities to identify the frost events triggering exceptional yield decreases.

### The Application of a Process-Based Simulation Model at Municipality Level

An underlying assumption of standard crop yield forecasting systems is that a point-based simulation model, originally developed to reproduce crop phenology and growth in a homogenous field (Jones et al., 2017), maintains its validity when executed in a gridded geographical domain, with a distinct set of input data per simulation unit. Here we apply the same rationale using the HAZEL model to simulate hazelnut yield across the main Turkish production regions. Further, we derive information from the statistical analysis of official yields to modulate the simulated growth processes, in order to increase model accuracy. Statistical techniques, such as simple or multiple linear regression, are commonly used in yield prediction systems (Sharif et al., 2017), both as a single method and as complementary tools to post-process the outputs of crop models (Lecerf et al., 2019). We used a long official series of yield data as the basis to infer the average reduction of the partitioning of assimilate to nuts, to allow reproducing the alternate bearing of hazelnut. Such an operation, which could be replaced by an explicit consideration of the carry-over effect on hazelnut growth in consecutive seasons (if such data will become available), demonstrated to be effective in increasing the model’s ability to reproduce the interannual variability of official yields.

The model calibration and evaluation proposed here would surely benefit from the availability of multi-year and multi-site field experimental datasets, in which crop phenological development and yield dynamics are monitored throughout the growing season (Bregaglio et al., 2020). An example of an ideal dataset needed to strengthen the calibration and evaluation of the HAZEL model is provided by Solar and Stampar (2011), who characterized the phenology, growing and yield capacity of 16 hazelnut varieties over 15 years of experiment in Slovenia. The availability of a similar dataset in Turkey would allow increasing the adherence of the model to reality, and in turn the performance of the whole prediction system. For instance, the differences in phenological development and yield potential of the Turkish hazelnut varieties have been not taken into account in the model parameterization. It is well known that the current genotypes have specific phenological traits, but sufficient information on the spatial distribution of the different varieties was not available. Nowadays, about 18 hazelnut cultivars are grown in Turkey (Erdogan, 2018), even though only four round shaped cultivars (Tombul, Palaz, Foşa, and Çakıldak) are the most relevant (Ayfer et al., 1986). Tombul is the most cultivated variety, which is, despite its identification, considered as a cultivar for marketing purposes, since many different clones are sould under the name “Tombul.”

Bostan (2009) investigated the appearance of male and female flowers, ovary growth and fruit formation and bud break in four main Turkish varieties and concluded that the main factors explaining the differences in cluster formation and leaf fall period are related to climatic variability and altitude. Despite the lack of information to develop variety-specific parameter sets, our results indicate that hazelnut phenological observations were reproduced with sufficient accuracy, in line with other modeling studies performed on tree species (Basler, 2016).

### Machine Learning Techniques in Crop Yield Forecasting

Crop yield prediction systems relying on process-based simulation models have flourished in the last decades, and were implemented as software frameworks (e.g., Thorp and Bronson, 2013; Shelia et al., 2019) or integrated modeling systems at various degrees of complexity (e.g., GEPIC, Liu et al., 2007; SMILE, Enders et al., 2010). Operational yield prediction systems are currently in use by international organizations, such as the Food and Agriculture Organization of the United Nations (AgroMetShell, Mukhala and Hoefsloot, 2004), and by governmental institutions, such as the European Commission (EU, MARS/BioMA, Donatelli et al., 2012; van der Velde and Nisini, 2019). The implementation of machine learning techniques in traditional crop yield forecasting systems has not yet become a standard, although recent realizations have already been the subject of review papers (Elavarasan et al., 2018; van Klompenburg et al., 2020). HADES embraces this trend and adopts an ensemble-based supervised learning algorithm, RF, in regression mode on top of a process-based modeling layer. RF is one of the most used machine learning techniques in agriculture thanks to its non-parametric nature, high predictive ability, internal evaluation of attributes, robustness to noise, and lack of proneness to overfitting (Rupnik et al., 2019). To date, applications of RF for yield prediction targeted, among others, mango (Fukuda et al., 2013) and switchgrass (Tulbure et al., 2012), whereby Jeong et al. (2016) proved that RF outperformed multiple linear regression for yield prediction of staple food crops at the regional and global level. In our study, other than being essential in improving system performances, RF provided a robust ranking of the predictors, confirming the added value of the process-based model application, whose simulated yields were top-ranked, as well as the relevant contribution of ground information on the targeted hazelnut systems.

### Outlook

HAzelnut yielD forEcaSt, as presented here, was executed considering the entire agro-meteorological time series from the start of each growing season until harvest. Such a system cannot be applied in an operational context, where yield predictions need to be available weeks to months before harvest. Future developments of HADES will focus on its accuracy in predicting official yields at defined timesteps prior to harvest, using seasonal or sub-seasonal weather forecast to complement the agro-meteorological time series until the end of the growing season (e.g., Hansen and Indeje, 2004). Furthermore, the cold stress function implemented in HAZEL (Supplementary Material 2), which allows capturing late frost events contributing to yield reduction in Turkey, should also be used as additional predictor in HADES. Also, the HADES implementation would encompass the consideration of other factors reducing the quantity and quality of hazelnut yield in the area, such as fungal diseases (Arciuolo et al., 2020) and viruses (Apple Mosaic Virus, Akbaş and Deǧirmenci, 2009).

Further developments will also employ the use of an ensemble of machine learning algorithms, whose development and applications are booming across disciplines, e.g., medicine (Peng, 2006) and economics (Zhu et al., 2016). This will allow inferring synthetic metrics from the distributions of the ensemble predictions, as recently done with crop models (Wallach et al., 2016), as well as evaluating differences in algorithms performance in specific conditions. The availability of free statistical packages implementing several machine learning algorithms to be run in ensemble mode (e.g., caret, Kuhn, 2008) will facilitate this operation and will be considered in the near future. In parallel to this, Everingham et al. (2016) demonstrated that subsetting the entire set of predictor variables used as input to a machine learning model to consider only the most important predictors as selected through RF variable importance can lead to more accurate yield predictions. This procedure can be extended from a single machine learning model to the entire ensemble of models.

Eventually, the performance of HADES will be tested in other regions of the world, which offers the possibility to assess the flexibility of the proposed system to predict yield in different environments that offer a diverse range of available predictors at different scales.

## Conclusion

In recent years yield prediction systems for tree crops have started to emerge in the landscape of agricultural modeling. Process-based crop simulation models and machine learning techniques have both been applied to different tree crops. However, so far, these two approaches have been mostly used independently from one another. HADES represents a new frontier in crop yield prediction, where process-based modeling and machine learning techniques are hybridized to enhance system performance. Each component of the system keeps the possibility to be either integrated in a single workflow or used stand-alone, thus fostering its implementation without affecting the whole system, e.g., when new data or models are available. The case study presented here is located in Turkey, the main hazelnut producing country, but HADES is scalable to any region of the world, where sufficient data are available. We deliberately considered a case study that covers a rather vast area, where data availability is relatively limited with several types of information being difficult to obtain due to the large spatial scale. The possibility to transfer the HADES system to any location of interest is promising for multiple stakeholders in the hazelnut sector that urgently needs predictive models applicable to different scales and in different regions.

## Data Availability Statement

The original contributions presented in the study are included in the article/Supplementary Materials, further inquiries can be directed to the corresponding author/s.

## Author Contributions

SB, KF, and LG conceived the HADES conceptual framework and wrote the manuscript with the support of FG. TV, FG, and SB performed the yield analysis and calibrated and evaluated the HAZEL model. KF and LG developed the machine learning layer. LG supervised the project. All authors contributed to the article and approved the submitted version.

## Conflict of Interest

KF and LG were employed by the Hazelnut Company division of Ferrero Trading Lux. The remaining authors declare that the research was conducted in the absence of any commercial or financial relationships that could be construed as a potential conflict of interest.

## Footnotes

1 https://power.larc.nasa.gov/
