# Supplementary material for: The HADES Yield Prediction System – A Case Study on the Turkish Hazelnut Sector
Source: Front Plant Sci. 2021 Jun 7;12:665471. doi: 10.3389/fpls.2021.665471 (PMC8216502; doi:10.3389/fpls.2021.665471)
Supplement: Supplementary file 1 [file Data_Sheet_1.docx]

Supplementary Material

# S1. HAZEL parameters after calibration

The HAZEL model parameters after calibration are shown in Table S1.1. The phenological model of hazelnut vegetative and reproductive phases presented in Bregaglio et al. (2016, 2020) was updated according to the new assessment scale in use by the agronomists who performed the field observations (Table S2).

Table S1.1. Phenological phases sampled in the Turkish orchards. These phases were reproduced in the HAZEL model according to the parameters presented in Table S1.2.

| Type | Phase | Description |
| --- | --- | --- |
| Reproductive (male flowers) | R1 | Catkins emerge |
|  | R2 | Catkins full developed but dormant |
|  | R3 | Catkins begin to elongate |
|  | R4 | Catkins in full bloom |
|  | R5 | Catkins withering |
| Reproductive (female) | R7 | Beginning of female flowering |
|  | R8 | Inflorescence in full bloom |
|  | R9 | End of female flowering |
|  | R10 | Ovaries develop |
|  | R11 | Cluster visible |
|  | R12 | Immature fruits |
|  | R13 | Fruits maturation |
|  | R14 | Fruits dropping |
|  | R15 | All nuts dropped |
| Vegetative | V1 | Dormant bud |
|  | V2 | Swollen bud |
|  | V3 | Budbreak |
|  | V4 | Leaf emergence |
|  | V5 | 3^rd^ leaf unfolded |
|  | V6 | Mature leaves are spotted |
|  | V7 | Mature leaves |
|  | V8 | Leaf senescence |
|  | V9 | Leaf shedding |
|  | V10 | Leaves dropping |

Table S1.2 Acronym, unit and description of the HAZEL model parameters calibrated in West and East Turkish hazelnut areas. Given that simulations were performed without considering water stress, the corresponding parameters were not used.

| Parameter | Description | Unit | Calibration | West | East |
| --- | --- | --- | --- | --- | --- |
| BaseTemperaturePhenology | Base temperature for phenological development | °C | N | 7 | |
| BranchVol | Branch to crown volume ratio | m^3^ m^-3^ | Y | 2.88E-04 | 2.92E-04 |
| Chilling_Threshold_Buds | Chilling hours to start thermal accumulation (buds) | h | Y | 321 | |
| Chilling_Threshold_Catkins | Chilling hours to start thermal accumulation (catkins) | h | Y | 318 | |
| Chilling_Threshold_Female | Chilling hours to start thermal accumulation (female) | h | Y | 350 | |
| Vcmax | Maximum carboxylation rate | micromoles m^-2^ s^-1^ | N | 28 | |
| FastSen | Daily amount of leaf senescence during leaf fall | kg C d^-1^ | N | 0.02 | |
| GrowthEff | Cost of growth respiration | unitless | N | 0.7 | |
| ImbThr | Imbalance threshold | unitless | N | 0.61 | |
| iniCabove | Tree initial aboveground carbon fraction | unitless | N | 0.5 | |
| iniCrown | Tree initial crown base height | m | Y | 0.34 | 0.28 |
| iniRadius | Tree initial crown radius perpendicular to row | m | Y | 2.88 | 3.15 |
| iniHeight | Tree initial height | m | N | 3 | |
| LeafDens | Leaf area density | m^2^ m^-3^ | N | 2.15 | |
| LeafC | Leaf carbon content | kg C kg^-1^ | N | 0.5 | |
| LeafMass | Leaf mass area | kg m^-2^ | N | 0.1 | |
| LeafSen | Daily amount of leaf senescence | kg C d^-1^ | N | 0.0015 | |
| LightCoeff | Coefficient for light response function | unitless | N | 0.01 | |
| NSCfract | Maximum non structural carbon allocated to growth | % | N | 0.03 | |
| FruitRip | Maximum partitioning to fruits | unitless | Y | 0.24 | 0.26 |
| RootDepth | Maximum root depth | m | N | 0.85 | |
| CondTmax | Maximum temperature for stomatal conductance | °C | N | 35 | |
| PhotoTmax | Maximum temperature for photosynthesis | °C | N | 35 | |
| MaximumTemperaturePhenology | Maximum temperature for phenological development | °C | N | 25 | |
| MaxDailyNSC | Maximum daily biomass from non structural carbon | % | N | 0.1 | |
| CondTmin | Minimum temperature for stomatal conductance | °C | N | 4 | |
| PhotoTmin | Minimum temperature for photosynthesis | °C | N | 4 | |
| CondTopt | Optimum temperature for stomatal conductance | °C | N | 25.5 | |
| PhotoTopt | Optimum temperature for photosynthesis | °C | N | 25.5 | |
| OptimumTemperaturePhenology | Optimum temperature for phenological development | °C | N | 18 | |
| LeavesRip | Partitioning to leaves at bud burst | unitless | Y | 0.55 | 0.68 |
| PARtoGlobalSolarRadiationRatio | PAR to global solar radiation ratio | unitless | N | 0.5 | |
| R1_Chill_Threshold | Chilling hours to reach phenological phase R1 | h | Y | 233 | |
| R2_Chill_Threshold | Chilling hours to reach phenological phase R2 | h | Y | 355 | |
| R3_Chill_Threshold | Chilling hours to reach phenological phase R3 | h | Y | 912 | |
| R4_Chill_Threshold | Chilling hours to reach phenological phase R4 | h | Y | 1085 | |
| R5_Chill_Threshold | Chilling hours to reach phenological phase R5 | h | Y | 1193 | |
| R7_Chill_Threshold | Chilling hours to reach phenological phase R7 | h | Y | 172 | |
| R8_ Chill_Threshold | Chilling hours to reach phenological phase R8 | h | Y | 375 | |
| R9_Chill_Threshold | Chilling hours to reach phenological phase R9 | h | Y | 704 | |
| R10_GDD_Threshold | Growing degree days to reach phase R10 | °C d^-1^ | Y | 95 | |
| R11_GDD_Threshold | Growing degree days to reach phase R11 | °C d^-1^ | Y | 339 | |
| R12_GDD_Threshold | Growing degree days to reach phase R12 | °C d^-1^ | Y | 732 | |
| R13_GDD_Threshold | Growing degree days to reach phase R13 | °C d^-1^ | Y | 786 | |
| R14_GDD_Threshold | Growing degree days to reach phase R14 | °C d^-1^ | Y | 1050 | |
| R15_GDD_Threshold | Growing degree days to reach phase R15 | °C d^-1^ | Y | 1981 | |
| Q10 | Relative respiration rate per 10° temperature increase | unitless | N | 2 | |
| BranchResp | Relative maintenance respiration of branches | g C g^-1^ d^-1^ | N | 0.002 | |
| RootFResp | Relative maintenance respiration of fine roots | g C g^-1^ d^-1^ | N | 0.002 | |
| LeavesResp | Relative maintenance respiration of foliage | g C g^-1^ d^-1^ | N | 0.01 | |
| FruitsResp | Relative maintenance respiration of fruits | g C g^-1^ d^-1^ | N | 0.01 | |
| StemsResp | Relative maintenance respiration of stems | g C g^-1^ d^-1^ | N | 0.002 | |
| RootHalfLife | Root half life | d | N | 250 | |
| SLAmax | Maximum specific leaf area | m^2^ kg^-1^ | Y | 20.8 | 21.2 |
| SLAmin | Minimum specific leaf area | m^2^ kg^-1^ | N | 8 | |
| CondTMax | Maximum stomatal conductance | °C | N | 0.65 | |
| NSCtarget | Non-structural carbon fraction target | % | N | 0.1 | |
| V1_GDD_Threshold | Growing degree days to reach phase V1 | °C d^-1^ | Y | 28 | |
| V2_GDD_Threshold | Growing degree days to reach phase V2 | °C d^-1^ | Y | 70 | |
| V3_GDD_Threshold | Growing degree days to reach phase V3 | °C d^-1^ | Y | 82 | |
| V4_GDD_Threshold | Growing degree days to reach phase V4 | °C d^-1^ | Y | 94 | |
| V5_GDD_Threshold | Growing degree days to reach phase V5 | °C d^-1^ | Y | 158 | |
| V6_GDD_Threshold | Growing degree days to reach phase V6 | °C d^-1^ | Y | 318 | |
| V7_GDD_Threshold | Growing degree days to reach phase V7 | °C d^-1^ | Y | 808 | |
| V8_GDD_Threshold | Growing degree days to reach phase V8 | °C d^-1^ | Y | 1661 | |
| V9_GDD_Threshold | Growing degree days to reach phase V9 | °C d^-1^ | Y | 3017 | |
| V10_GDD_Threshold | Growing degree days to reach phase V10 | °C d^-1^ | Y | 4091 | |
| CondVPDmax | Vapor pressure deficit for maximum conductance | KPa | N | 0.2 | |
| CondVPDmin | Vapor pressure deficit for minimum conductance | KPa | N | 3.7 | |
| WoodC | Wood carbon content | kg C kg^-1^ | N | 0.5 | |
| WoodDens | Wood volumetric mass density | kg m^-3^ | N | 750 | |

# S2. Considering the effect of frost in reducing hazelnut yields

The effect of extreme frost events in reducing hazelnut yield has been implemented in HAZEL according to the algorithm presented in Figure S2.1.

Supplementary Figure S2.1. Workflow of the algorithm developed to simulate the effect of cold stress on hazelnut yield

When temperature is below 0, a cold stress function (*F_cold_*, 0-1) in the form of an inverse logistic driven by hourly air temperature is computed (Eq. 1, Fig S2).

$F_{cold}=\frac{1}{\left. 1+exp (-k\left( -Tair-lag \right) \right)}$ [Eq.1]

where k (1.55) and lag (4) are empirical coefficients, set in agreement with the experimental data from (Chozinski, 1994) and Tair is hourly air temperature (°C).

Figure S2.2. Cold stress function used to consider the impact of extreme frost events on hazelnut yield.

The function starts to consider a frost event when temperature drops below 0, with a smooth increase until -2°C. Then, an almost linear increase of the cold stress function is considered up to -6°C. Air temperatures below these thresholds are considered as extremely dangerous for hazelnut fruits (*F_cold_* > 0.8). The increased sensitiveness of hazelnut fruits during reproductive development is computed according to the function drawn in Figure S2.3.

Figure S2.3. Function used to reproduce the sensitivity of hazelnut fruits to cold stresses.

The sensitivity to cold stress is 0 until mid-phase R9 (End of flowering), then it linearly increases up to R12 (immature fruits), when it reaches the maximum. This function mimics the larger impact of late cold stress on hazelnut fruits.

At the end of the day, the cumulated value of *F_cold_* multiplied by the sensitivity function are translated into a percent reduction of daily partitioning to fruits (Str_cold_, 0-1), according to Eq. 2:

$DailyPart=MaxPart \times(1-{Str}_{cold})$ [Eq.2]

Where *DailyPart* is the daily partitioning to hazelnut fruits (unitless, 0-1), *MaxPart* is the maximum partitioning to hazelnut fruits (model parameter, unitless, 0-1).

# S3. Probability distribution of alternate bearing intensity index

Figure S3.1 presents the probability distributions of alternate bearing intensity index computed after bootstrap resampling with replacement (5000 samples) in synthetic yield series for each province of West and East Turkish hazelnut producing area.

 Fig. S3.1. Probability distribution of alternate bearing intensity values (*I*) obtained from sample permutations. The *I* value of the original series is reported with a blue line, the intensity value below which the interannual yield variation was considered as negligible (*I_0.001_*) with a green line. Plots also report the significance of the original *I* (*p_(I)_*).

**S4. Probability distribution of alternate bearing intensity index**

Figure S4.1 presents a detailed comparison of model performance on the level of the main hazelnut growing municipalities by means of R² and MAE sampling distributions that resulted from predicting official yields over all 100 bootstrap samples. Median model performances are depicted with vertical dashed lines (blue in calibration, red in validation). In terms of both R² and MAE, median model performance was slightly better in validation (R^2^ = 0.75, MAE = 0.14 t ha^-1^) underlining the robustness of the presented procedure to overfitting. For both metrics, standard deviation was generally small in calibration as well as in validation, with values ranging between 0.01 t ha^-1^to 0.02 t ha^-1^ for MAE and 0.03 to 0.06 for R².


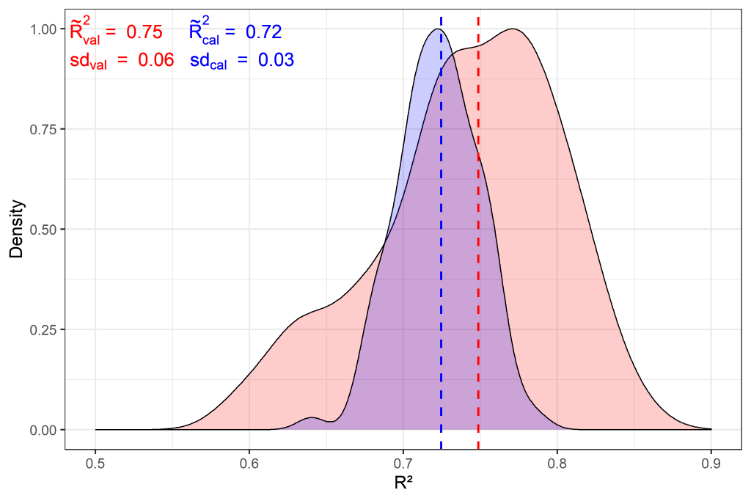


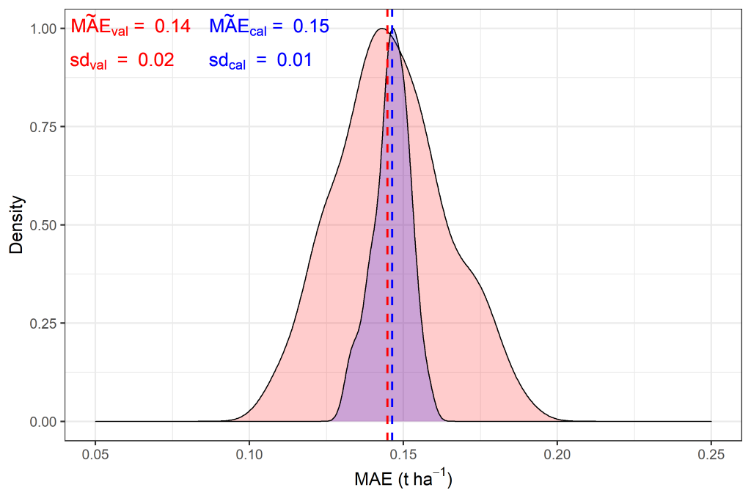


Figure S4.1. Comparison of HADES performance in predicting official yield in terms of R² (top) and mean absolute error, MAE (bottom) for the main hazelnut growing municipalities. The drawn sampling distributions result from 100 bootstrap samples. Cross-validated model performance in calibration (cal) is shown in blue, performance in validation (val) in red. The median values of the respective distributions are shown as dashed lines. sd = standard deviation.
